# Latency to and duration of desensitization following cornual nerve blocks of 3 local anesthetics in goat kids

**DOI:** 10.3168/jdsc.2026-1089

**Published:** 2026-08-06

**Authors:** Erica Schmidt, Alonso G.P. Guedes, Beth A. Ventura, Gerard Cramer, Whitney A. Knauer

**Affiliations:** 1 Department of Veterinary Population Medicine, College of Veterinary Medicine, University of Minnesota, Saint Paul, MN 55108; 2 Department of Veterinary Clinical Sciences, College of Veterinary Medicine, University of Minnesota, Saint Paul, MN 55108; 3 Department of Large Animal Clinical Sciences, College of Veterinary Medicine, Michigan State University, East Lansing, MI 48824

## Abstract

Local anesthetic nerve blocks are widely used to reduce pain during the disbudding procedure. In dairy calves, lidocaine is the standard choice because it is fast-acting and inexpensive. However, in goat kids, lidocaine's efficacy is constrained by its short-acting duration, narrow safety margin, and challenges in administration. Longer-acting anesthetics have been shown to improve pain control in other species. Our objective was to describe the latency to and duration of desensitization for buffered lidocaine (BL), bupivacaine (BUP), and bupivacaine liposome injectable suspension (BLIS) for a cornual nerve block in goat kids. Twenty-four kids (5 ± 2 d old) met study inclusion and retention criteria and were randomly assigned to one of 3 treatments (n = 8/group). A single horn bud was anesthetized with 1 mL of anesthetic divided equally between the cornual branches of the infratrochlear and lacrimal nerves. A nerve stimulator was used to assess baseline sensitivity before and at predefined intervals after treatment to assess latency to (2.5, 5, 6, 7, 8, 9, 10, 12.5, 15 min) and duration of (BL and BUP every 0.25 h; BLIS at 0.5, 1, 2, 4, 6, 8, 24, 48, 72 h) desensitization. All treatments achieved a complete cornual nerve block within 8 min after injection (BL: 5–6 min, BUP: 5–7 min, BLIS: 5–8 min), demonstrating a similar latency. The duration of desensitization varied among treatments. The BLIS lasted the longest (8–72 h), although the number of desensitized kids decreased over time (n = 8 at 6 h; n = 5 at 8 h; n = 1 between 24 and 72 h). In contrast, desensitization with BUP lasted 1.75 to 3.5 h (n = 8 at 1 h; n = 6 at 1.75 h; n = 4 at 2.25 h; n = 2 at 3.25 h), whereas BL offered the shortest period of desensitization, lasting 1 to 2.25 h (n = 6 at 1 h; n = 1 at 1.75 h). Our findings suggest that BLIS offers the greatest potential for extended pain relief without compromising the latency to desensitization.

## Graphical Abstract

**Figure:**
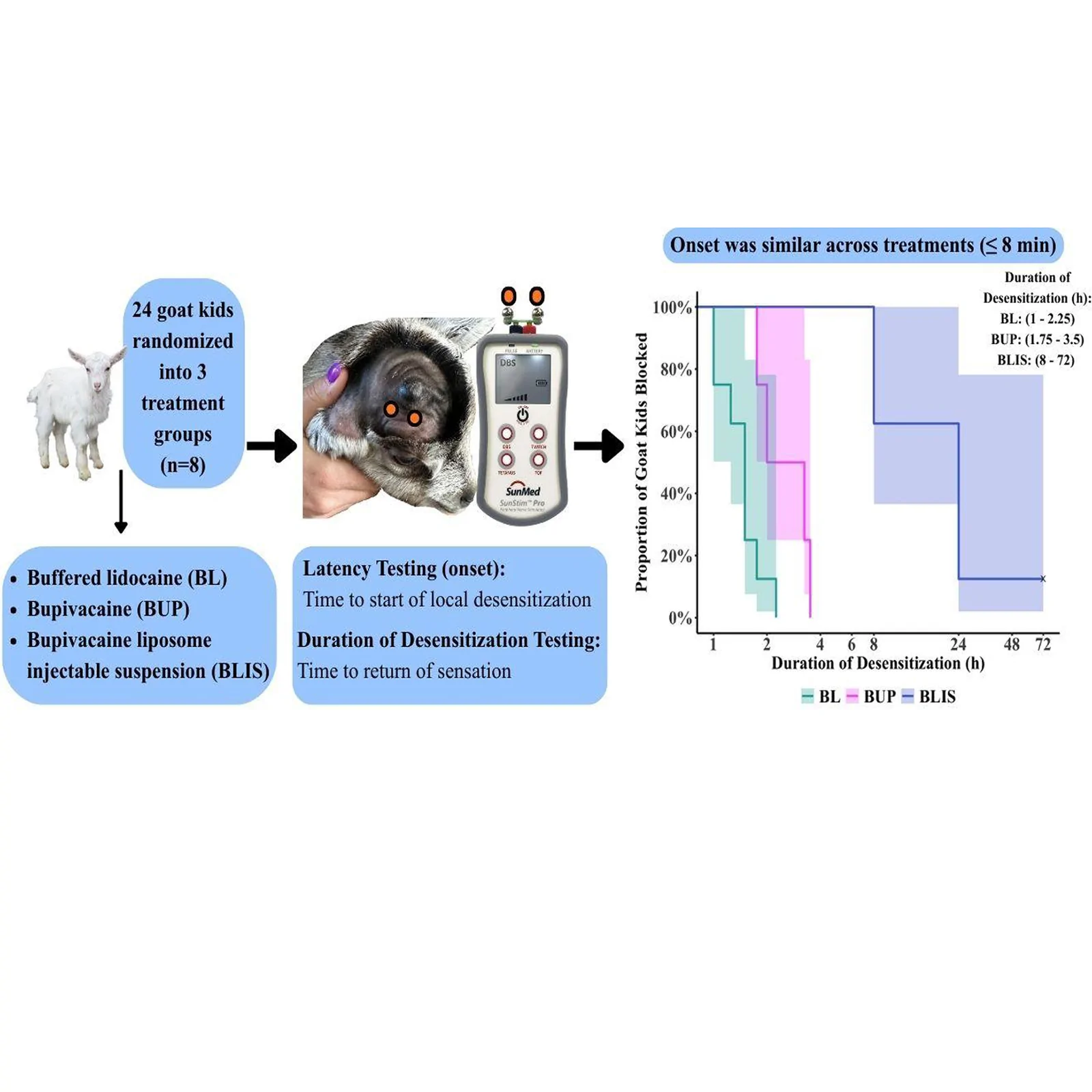

Cautery disbudding, or thermal destruction of horn-producing tissue, is a common management practice in goat production systems to improve herd safety and animal handling (USDA, 2020). Disbudding is well established as a painful procedure that causes acute and long-term pain in goat kids (Alvarez et al., 2009, 2019; Hempstead et al., 2017). Despite this routine husbandry procedure and evidence of pain, an estimated 70% of disbudded goat kids in the United States do not receive pain relief (Valdmanis et al., 2007; USDA, 2020). One potential explanation for this is that no standardized pain mitigation protocol for disbudding has been established, despite growing recognition of disbudding pain (Hempstead et al., 2019a; Singh et al., 2024). Furthermore, there is no US Food and Drug Administration (**FDA**)-approved product for pain management associated with disbudding in the United States, necessitating extra-label drug use in accordance with the Animal Medical Drug Use Clarification Act (AASRP, 2020).

Although injectable lidocaine (a short-acting local anesthetic) is commonly used in disbudding protocols for dairy calves due to its ability to reduce the physiological and behavioral indicators of pain associated with cautery disbudding (Stock et al., 2013), the efficacy of local anesthesia has been inconsistent in goat kids (Hempstead et al., 2019b). Some studies report a reduction in pain-related behaviors with lidocaine injected as a block in both the cornual branches of the lacrimal and infratrochlear nerves (Chandrahas et al., 2013; Alvarez et al., 2015), whereas others have found elevations in plasma cortisol similar to those of kids disbudded with no local block (Hempstead et al., 2020). Pain on injection caused by the low pH of lidocaine injectable solution (pH ∼4.5; Adcock and Tucker, 2022) has been suggested as an important confounding factor obscuring proper assessment of local anesthetic effects. Buffering lidocaine with sodium bicarbonate, a technique shown to reduce injection pain in humans (Matsumoto et al., 1994), has therefore been explored as a refinement (Knauer et al., 2023). However, although buffered lidocaine (**BL**) may reduce struggle behaviors and nociceptive sensitivity following disbudding, it does not appear to fully resolve the acute cortisol response to disbudding (Knauer et al., 2023).

Lidocaine's short duration of action also limits its effectiveness in goat kids, with head-directed behaviors emerging as early as 20 min after disbudding despite an effective nerve block (Venkatachalam et al., 2018). In contrast, dairy calves showed increased head-directed behaviors later, ∼50 to 75 min after disbudding (Adcock et al., 2020), suggesting that although lidocaine has a short duration in both species, its anesthetic effects may appear to dissipate sooner in goat kids than in calves. Longer-acting local anesthetics are therefore of interest to investigate.

Bupivacaine HCl (0.5%) has been shown to provide extended spinal anesthesia in adult goats (Fackler et al., 2024), yet its effectiveness has not been studied relative to disbudding of goat kids. More recently, an extended-release formulation, bupivacaine liposome injectable suspension (**BLIS**), has been FDA-approved in dogs (as a single dose infiltration to provide postoperative analgesia for cranial cruciate ligament surgery) and cats (as a peripheral nerve block to provide regional postoperative analgesia following onychectomy), providing postoperative analgesia for up to 72 h (Enomoto et al., 2016; Lascelles et al., 2016). In calves undergoing disbudding, BLIS administered as a cornual nerve block provided comparable pain relief to a combination of lidocaine and meloxicam based on mechanical nociceptive thresholds and behavioral measures (Martin et al., 2022), making it a promising candidate for further evaluation in goat kids.

Characterizing the latency to and duration of desensitization (i.e., time to onset and duration) is a critical first step in evaluating timing expectations and whether these drugs may be useful in refining disbudding protocols in goat kids. Therefore, the objective of this study was to provide a descriptive overview of the latency to and duration of desensitization of the horn bud produced by BL, bupivacaine (**BUP**), and BLIS in goat kids.

All study activities were conducted following ethical approval by the University of Minnesota Institutional Care and Use Committee (protocol #2412-42617A). This study took place from March to April 2025 on a commercial dairy goat farm located in Minnesota. Upon birth, goat kids were immediately separated from their dam and brought to a temperature-controlled room and placed in a bin (113-L plastic bins lined with newspaper) with their littermates according to the farm's existing protocols. All kids received 500 mL of heat-treated goat colostrum in 2 feedings within the first 24 h. Thereafter, kids received 2 daily feedings with dairy cow pasteurized whole milk at ∼20% body weight. At 3 d of age, kids were moved to a temperature-controlled barn, where they were housed in groups (4–6 kids/group) in raised plastic tubs measuring 3.3 m^2^. Kids remained under the management of the commercial farm for the duration of the experiment, apart from the testing procedure described in the following paragraphs.

Electrical stimulation delivered by a peripheral nerve stimulator (**PNS**; SunStim Pro, SunMed), adapted from Fierheller et al. (2012), was used as the nociceptive method for local anesthetic validation. Because their protocol was designed for calves, a preliminary trial was conducted to optimize effectiveness and species-specific suitability. Electrode type, placement, and stimulation parameters were defined in preliminary testing (phase 1) using 10 healthy goat kids between 3 and 7 d of age (Alpine n = 5, Saanen n = 4, and Toggenburg n = 1; doelings n = 6 and bucklings n = 4) over a 3-d period. The hair surrounding the horn buds was clipped, and the skin was cleaned using 50% isopropyl alcohol solution. The kids were gently restrained on the lap of a trained assistant for measurements. Electrode type included ball-type and electrocardiogram (**ECG**) electrodes (3M Red Dot infant monitoring), as shown in Figure 1, and stimulation modes included double burst stimulation (**DBS**; two 50-Hz stimuli delivered 75 ms apart), twitch (stimulus delivered at 1 Hz until button is released), and train of 4 (TOF; 4 equal-intensity stimuli 30 ms apart), with increasing current intensities from 10 to 70 mA or until a clear aversive response (see Table 1 for ethogram) was elicited, whichever came first.

**Figure 1:**
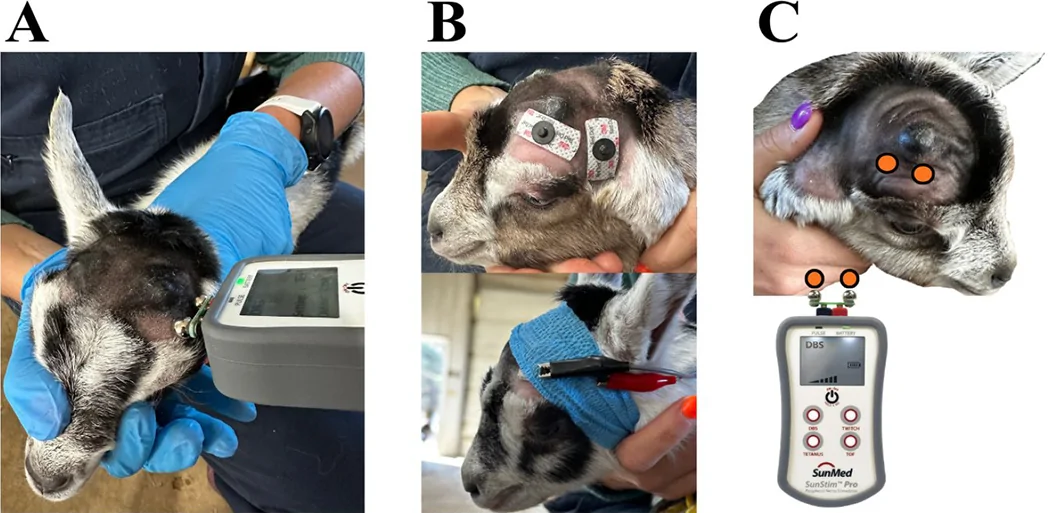
Electrode configurations used during phase 1 to refine cornual nerve stimulation. (A) Refined ball electrode placement targeting presumed locations of the cornual nerve branches. (B) Modified ECG electrodes cut into strips to more precisely target the cornual nerves. (C) Final ball electrode placement (indicated by orange circles) to target the cornual nerve injection sites for assessment of baseline sensitivity and evaluation of anesthetic latency to and duration of desensitization.

**Table 1:** Ethogram of observed behaviors from nerve stimulation developed during phase 1^1^

Behavior	Description
Body flinch	A slight, brief contraction of the body characterized by mild torso tightening or a small sudden jolt.
Ear flick	Rapid movement of one or both ears to the front and back, independent of a head shake.
Escape	Forward or backward movement of the whole body away from the point of stimulation, often involving pushing or stepping with the legs.
Head shake	Rapid, repeated tilting of the head side to side from stimulation.
Head withdrawal	Rapid lateral movement of the head away from the stimulator following stimulation.
Skin flinch	Localized contraction of the skin surrounding the horn bud in response to stimulation.
Vocalization	Emission of a bleat with either an open or closed mouth occurring during or immediately following stimulation.

1 Selected definitions were adapted from Knauer et al. (2023), whereas additional behaviors were defined for this study. These behaviors formed the basis used to determine latency to and duration of desensitization produced by local anesthetics in goat kids.

Initial testing revealed that both the ball-type and ECG electrodes produced inconsistent behavioral responses, which were attributed to poor skin contact or nonspecific nerve stimulation (n = 4 kids). Refining the ball electrode placement between the cornual branches of the lacrimal and infratrochlear nerves (Figure 1A) and modifying ECG electrodes into narrow strips targeting both branches (Figure 1B) improved targeting (n = 4 kids). Results showed that DBS at 40 to 70 mA was the most reliable mode to elicit aversive behavioral responses. The final validation phase, which involved a BL block to confirm desensitization with needle prick mapping in 2 kids, showed that clear aversive responses were still elicited by the ECG electrodes. This outcome suggests the electrodes may have been too large, potentially stimulating nerves adjacent to the anesthetized area. Consequently, the ball electrode configuration (Figure 1C), paired with DBS, was selected for the subsequent local anesthetic validation (phase 2, below). We opted to apply stimulation to an individual horn bud due to practical considerations: the design of the ball electrode allows for stimulation of only one bud at a time and would subject the kids to unnecessary, repeated electrical stimulation.

To be eligible for inclusion in phase 2, kids needed to be healthy (evaluated using a goat kid health scoring system, Menn et al., 2023), less than 14 d in age, and of the Alpine or Saanen breed. Both doelings and bucklings were eligible for enrollment. Thirty-three kids (22 Alpine; 11 Saanen) met these criteria and were enrolled. Kids entered the study on a rolling basis (n = 3 per day) and were randomly assigned to receive a cornual nerve block with either BL (n = 10), BUP (n = 9), or BLIS (n = 14). Treatment sheets were created by investigators using a computerized random number generator with no allocation concealment. The same investigators enrolled and assigned kids to treatments blocking by sex (male or female) and horn bud side (right or left) to ensure balanced allocation across groups over the course of the study. Hair was clipped around one horn bud (from above the eyes to below the temporal region) and the skin was cleaned with 50% isopropyl alcohol. Body weight (kg) at enrollment was collected.

Before treatment, baseline stimulation thresholds eliciting a clear aversive behavioral response were determined for each kid using the ball-type electrode and DBS stimulation. Throughout the study, 3 different trained assistants were responsible for restraining goat kids while measurements were taken. They provided gentle manual restraint to the kids, making sure their eyes were covered and their ears were positioned ventrally to prevent interference with the PNS. Behavioral responses were assessed by a non-blinded assessor using a scale based on the ethogram outlined in Table 1: grade 0 = no reaction (no visible change in posture, movement, or expression); grade 1 = mild reaction (body flinch, ear flick, or skin flinch); grade 2 = strong reaction (escape, head shake, head withdrawal, or vocalization). For each kid, stimulation current that elicited a grade 2 behavioral response was recorded and used to determine local anesthetic latency to and duration of desensitization.

Each enrolled goat kid received an injection over the cornual branches of the lacrimal and infratrochlear nerves on one randomly assigned horn bud. Buffered lidocaine was prepared by mixing 1 mL of 8.4% sodium bicarbonate (VetOne, MWI, Boise, ID) with 9 mL of 2% lidocaine (VetOne, MWI, Boise, ID) in a sterile red-top tube, yielding an 18 mg/mL lidocaine solution. Bupivacaine hydrochloride consisted of a commercial 0.5% solution (5 mg/mL, Xellia Pharmaceuticals, Buffalo Grove, IL; manufactured by Steriscience Specialties Pvt Ltd., Bengaluru, India), and BLIS consisted of a 13.3 mg/mL liquid suspension (Nocita; Elanco USA Inc., Greenfield, IN). Contrary to the label recommendations, the initial protocol for administering BLIS to the first 4 goat kids in the treatment group involved multiple needle entries to the vial. This was later corrected to a single needle (20-gauge × 1-in [2.5 cm]) entry followed by drawing multiple doses into the same syringe. A veterinarian performed a 2-site subcutaneous injection (0.5 mL/site) of the anesthetic solution targeting the cornual branches of the lacrimal and infratrochlear nerves using a 22-gauge × 3/4-in (1.9 cm) needle and a 3-mL syringe.

Assessments were performed at 2.5, 5, 6, 7, 8, 9, 10, 12.5, and 15 min after injection. Latency to desensitization was defined as a grade 0 behavioral response, with the block considered unsuccessful if a grade 1 or 2 behavioral response persisted at 15 min because any sensation indicates incomplete desensitization. In these cases, a needle prick was used to verify that the observed reaction indicated a true lack of desensitization, and the animal was excluded from further testing (n = 5). Duration of desensitization was defined as the time from onset to resumption of grade 2 behavioral response. Although a grade 1 response indicated the anesthetic was beginning to wear off, a grade 2 response was required to confirm a complete return to baseline sensitivity. This was measured at intervals based on the expected duration of each anesthetic. Accordingly, animals treated with BL and BUP were evaluated every 0.25 h, whereas those treated with BLIS were assessed at extended intervals of 0.5, 1, 2, 4, 6, 8, 24, 48, and 72 h after injection. Testing concluded when a return of the grade 2 response was observed or at 72 h, whichever came first. Behavioral responses to nerve stimulation (grade 0, 1, 2) were observed and recorded live by the same trained individual who applied the ball electrode and delivered the stimulation, ensuring consistency in both procedure and behavioral assessment throughout the study. Stimulation would be repeated to confirm behavioral responses when initial reactions were unclear or ambiguous. Assessments were not blinded, as this was a descriptive study; the same team applied treatments and performed assessments.

This study was designed to give a descriptive and practical overview of latency to and duration of desensitization for 3 local anesthetic treatments in goat kids, and therefore no formal sample size calculation was conducted. Enrollment of kids was a convenience sample based on the availability of kids at our study farm, resulting in a final study sample size of 24 kids (8/group) following exclusions from the initial 33 enrolled. This is comparable to that used in a similar calf study (Fierheller et al., 2012).

Baseline characteristics (age, weight, sex [male or female], horn bud [right or left], and breed [Alpine or Saanen]) of enrolled goat kids were summarized across treatment groups. Continuous variables were summarized using means, standard deviations, and ranges; categorical variables were summarized as counts. Data were analyzed using RStudio (v 9.1.401, Posit team 2025) and R (v 4.3.3, R Foundation for Statistical Computing, Vienna, Austria).

Latency to and duration of desensitization were analyzed as time-to-event outcomes. These outcomes were visualized using Kaplan–Meier survival curves. The range for both outcomes was reported for each treatment group, along with the number of kids remaining desensitized (at risk) at comparable time points. Animals who were still desensitized at the last evaluation time point (72 h) were right-censored at that time. Given the descriptive nature of this study, these analyses were considered exploratory to support interpretations. No other analyses were performed.

Of the 33 kids eligible for testing, 5 were excluded for failing to meet retention criteria (BL, n = 2 [doeling right bud; doeling left bud]; BUP, n = 1 [buckling right bud]; BLIS, n = 2 [buckling left bud, doeling right bud]). Of the remaining 28 kids, 4 kids initially assigned to the BLIS treatment were removed due to drug-handling issues unrelated to retention criteria and were replaced with 4 new kids, which were included in the final sample. The final analysis consisted of 24 kids (Alpine: n = 15 [BL = 4, BUP = 6, BLIS = 5]; Saanen: n = 9 [BL = 4, BUP = 2, BLIS = 3]). Mean ± SD (range) age was 5 ± 2 (2–14) d and weight was 4.1 ± 0.5 (2.6–5.2) kg. Age and weight were comparable across treatment groups.

Latency to desensitization was similar across treatment groups (range): BL (5–6 min), BUP (5–7 min), and BLIS (5–8 min; Figure 2a). By 8 min, all kids (n = 24) achieved a grade 0 behavioral response. In contrast, the duration of desensitization varied across treatment groups (Figure 2b). Following BLIS treatment, desensitization lasted between 8 and 72 h. All goat kids in the BLIS group remained desensitized at 6 h, with 5 of the 8 kids maintaining this state at 8 h. Notably, one kid remained desensitized through 72 h. In contrast, desensitization did not persist beyond 2.25 h for the BL group or 3.5 h for the BUP group. At 1.75 h, 6 of 8 kids in the BUP group remained desensitized compared with 1 of 8 in the BL group. At 2.25 h, 4 of 8 BUP kids remained desensitized, and by 3.25 h, only the last 2 kids in the BUP group retained desensitization.

**Figure 2:**
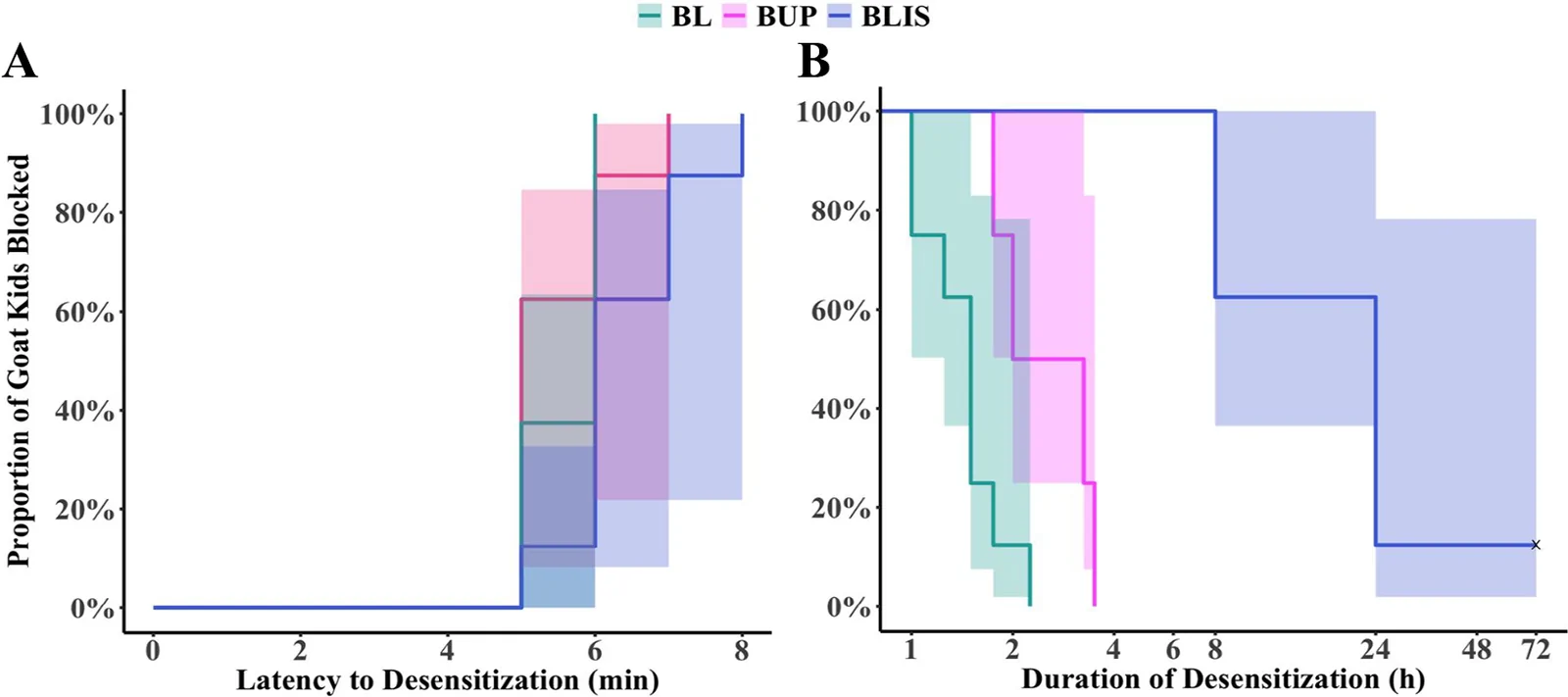
Kaplan–Meier plots illustrating latency to desensitization (A) and duration of desensitization (B) of cornual nerve blocks in goat kids that met study retention criteria following injection of BL (n = 8), BUP (n = 8), or BLIS (n = 8). Censored observations in panel B at 72 h are denoted with “x.” The shaded areas represent the 95% CI for the survival probability estimate.

In our study, all kids across treatment groups achieved complete latency within 8 min, which is consistent with the known effects of each local anesthetic. For example, lidocaine's lower negative logarithm (base 10) of the acid dissociation constant (**pKa**) and buffering facilitates faster diffusion across neuronal membranes, resulting in a rapid onset, whereas the higher pKa of BUP is associated with an intermediate onset (Best et al., 2015; Taylor and McLeod, 2020). Although the latency of BLIS was slightly delayed, kids still showed desensitization within a short period, similar to that observed with BUP. Data on the onset of BLIS in veterinary species are currently limited, although human studies report an onset of 2 to 5 min for BLIS, which is comparable to BUP (Apseloff et al., 2013).

All local anesthetics tested provided a duration of desensitization that surpassed the time required for disbudding, confirming that the immediate acute pain associated with the procedure should be expected to be effectively blocked. However, the duration of the effect varied among treatments, reflecting the anticipated duration of each drug. Lidocaine's short half-life is attributed to its lower lipid solubility and moderate protein binding (Beecham et al., 2025). In goat kids, its duration is further limited by a narrow therapeutic margin, with a maximum recommended dose of 7 mg/kg (Venkatachalam et al., 2018). The relatively short duration induced by BL, in which all kids regained sensation within 2.25 h, may be attributed to the cumulative effect of these factors. Bupivacaine maintained moderate duration, with all kids regaining sensation by 3.5 h. Increased potency relative to lidocaine, coupled with greater lipid solubility and higher protein binding (∼95%), likely accounts for its longer duration of action (Malik et al., 2017; Shafiei et al., 2025). This aligns with findings in adult goats, in which lumbosacral injection of 0.5% bupivacaine resulted in motor blockade for over 3 h, with a median time to walk of ∼225 min (Fackler et al., 2024). Finally, BLIS provided the longest period of desensitization: all kids remained desensitized for at least 6 h, most kids regained sensation between 8 and 24 h, and one kid remained blocked for 72 h. Despite BLIS being labeled to provide up to 72 h of pain relief in dogs and cats, clinical trials evaluating its efficacy have shown that ∼30% of dogs and ∼25% of cats assessed were still in pain within the first 24 h following surgery (FDA, 2016, 2018). Therefore, animals should be monitored for signs of pain rather than relying solely on the expected duration of the anesthetic. Furthermore, because this drug was labeled for dogs and cats and evaluated here in goats under different procedural conditions, the duration of effect may not be directly comparable across clinical contexts or species.

Pharmacological and procedural factors can both influence the duration of local anesthesia. In this study, goat kids did not undergo cautery disbudding, resulting in no tissue injury or inflammation, which may have allowed the anesthetic to persist longer than would be seen following a disbudding procedure. This is important because tissue injury and the associated inflammatory response may alter pharmacokinetics and pharmacodynamics. Specifically, inflammation-related vasodilation may increase local blood flow and promote faster clearance from the injection site (Wautier and Wautier, 2022; Garmon et al., 2026). Furthermore, species-related differences in drug metabolism are well documented: for example, goats (compared with cattle) have faster excretion of certain analgesics, such as meloxicam (de la Puente et al., 2024), suggesting that analgesic effects may dissipate sooner in goat kids as compared with calves, potentially affecting the success of pain mitigation. Further research is necessary to determine if a similar pattern exists for local anesthetics.

It is important to note that in our study, 15% of the kids failed to achieve an effective block regardless of treatment. In goat kids, this variability may be partly explained by anatomical differences affecting block success and latency. For example, an anatomical study of Black Bengal goats demonstrated breed and sex-based differences in the dimensions (length and breadth) of the caudo-frontal depression, a crucial anatomical landmark for this specific type of block (Poddar et al., 2025). We urge veterinarians to prioritize confirming the effectiveness of cornual nerve blocks in clinical practice, rather than relying exclusively on anatomical landmarks. We also suggest that additional research across a wider variety of goat breeds is necessary to fully understand any potential breed-specific variations.

Despite providing valuable insights, this study has limitations. Handling BLIS on farm was challenging, necessitating the re-enrollment of 4 additional kids. Initially, multiple needle entries were used, contrary to label recommendations. This was later changed to a single entry followed by drawing multiple doses into the same syringe, based on evidence that single-use BLIS vials can be used as multidose vials for up to 4 d if aseptic technique is maintained (Wang et al., 2021). However, maintaining asepsis on farm is difficult. This difficulty, combined with factors such as transport, temperature fluctuations, and the drug's viscosity, could have compromised the integrity of the liposomes. Second, though a standardized protocol was used, 3 different people assisted with restraint due to personnel availability. This may have introduced variability in handler technique that could have influenced kid behavior or electrode contact. Finally, we acknowledge that interobserver reliability was not formally assessed for the behavior observations, as the assessor who applied stimulation was aware of the expected block duration, introducing potential for bias despite the use of an ethogram and consistent criteria. Finally, the varying assessment intervals between treatments were essential to balance pharmacologic expectations, ensure the welfare of the goats by avoiding overly frequent shocks over an extended period, and maintain logistical feasibility. However, this reduced the precision of BLIS duration estimates, especially within the range of 8 to 24 h. Although our findings are useful for estimating disbudding procedure timing, larger studies with more goat kids are needed to draw stronger inferences when comparing anesthetic latency to and duration of desensitization.

Based on our findings, all 3 local anesthetic treatments evaluated provided rapid-onset (within 10 min) of cornual nerve blocks in neonatal goat kids while also appearing to persist long enough to cover the disbudding procedure. Our findings indicated that BLIS appeared to persist the longest compared with BUP and BL, supporting its potential application as a prolonged local anesthetic for procedures such as cautery disbudding in goat kids. However, it should be noted that regardless of anesthetic approach used, the duration of anesthesia is unlikely to cover the entire duration of pain associated with disbudding.
